# Strong Bodies, Stronger Bonds: The Intersection of Strength Training Intensity and Sexual Pleasure

**DOI:** 10.1192/j.eurpsy.2025.2317

**Published:** 2025-08-26

**Authors:** S. Ben Aissa, C. Najar, K. Razki, M. Cheour, F. F. Romdhane

**Affiliations:** 1 Razi Hospital, La Manouba, Tunisia

## Abstract

**Introduction:**

Strength training has long been linked to several physical and mental health advantages, ranging from increased muscle strength and endurance to higher mood and self-esteem. However, the potential link between strength training intensity and sexual satisfaction is a relatively unexplored area of research.

**Objectives:**

This study’s objective is to evaluate the potential relationship between the intensity of strength training and an individual’s degree of sexual satisfaction among people who exercise at the gym in Tunisia.

**Methods:**

This is a cross-sectional study, conducted from February to March 2024. Participants were recruited online through social media platforms (Tunisian facebook groups and fitness forums) using a posted survey link. We’ve included respondents who are 18 years of age or older who have been active in strength training with a gym membership for 1 month or more. The respondents were required to answer a questionnaire that included socio-demographic data and to provide strength training intensity related details (sessions frequency, duration, perceived overall intensity using likert scale)

Sexual satisfaction was measured using the Sexual Satisfaction Index (SSI), validated psychometric tool developed by Leth-Nissen et al. in 2021. The sum score of the SSI can range from 0 to 36. A higher sum score indicates a higher level of sexual satisfaction.

**Results:**

The total number of participants was 72, with 86% being male. The majority of responders (n=65, 90.2%) indicated that they performed strength training exercises at least three times per week, with an average session length of 45 minutes. In terms of strength training intensity, 38.8% (n= 28) of participants reported high-intensity sessions, 48.6% (n=35) moderate-intensity sessions, and the remaining participants reported low-intensity sessions.

Analysis of the Sexual Satisfaction Index scores revealed a mean score of 23.6 (SD = 6.2), indicating that individuals had moderate to high sexual satisfaction. A significant association was found between strength training intensity and sexual satisfaction scores (r = 0.42, p < 0.01), indicating that higher intensity exercises are associated with higher sexual satisfaction.

**Conclusions:**

Our findings aim to shed light on the link between fitness habits and sexual well-being, emphasising the potential value of including exercise interventions in talks about sexual health and satisfaction. The findings could indicate that the benefits of strength training go beyond physical fitness, potentially contributing to improved overall well-being. More research is needed to delve deeper into the underlying mechanisms causing this link and to investigate its broader implications for overall health and quality of life.

**Disclosure of Interest:**

None Declared

